# Post transplant thrombotic microangiopathy causing acute renal failure

**DOI:** 10.4103/0971-4065.53327

**Published:** 2009-04

**Authors:** S. Qutube, K. G. Arun, N. Jayaram, S. Ramakrishnan, R. Dilip

**Affiliations:** Department of Nephrology, NU Trust, Padmanabhanagar, Bangalore - 560 070, India; 1 Anand Institute of Laboratory Medicine, Blue Cross Chambers, Infantry Road Cross, Bangalore G.P.O., Bangalore - 560 001, Karnataka, India

**Keywords:** Acute renal failure, anti phospho lipid antibody syndrome, renal transplant, thrombotic micro angiopathy

## Abstract

Acute Renal Failure (ARF) in the immediate post transplant period is most commonly due to acute tubular necrosis, acute cellular rejection and calcineurin inhibitor toxicity apart from usual prerenal and post renal causes. In this report, we discuss an interesting and unusual cause of ARF due to thrombotic micro angiopathy in the immediate post transplant setting.

## Introduction

ARF in the post transplant period is most commonly due to acute tubular necrosis (especially in the deceased donor transplant recipients), acute cellular rejection and calcineurin inhibitor toxicity. Though not common, post transplant thrombotic micro angiopathy is being reported with increasing frequency worldwide; its incidence is particularly higher in the transplant patients than in the general population. In the setting of renal transplantation, it can appear for the first time (de novo) or can occur in recipients whose primary kidney disease was Hemolytic Uremic Syndrome (recurrence) (1). In this case report, we discuss the occurrence of this disease in a living related donor renal transplant recipient who had anti phospho lipid syndrome in the pre transplant period, the cause of which is not clear.

## Case Report

A 48 year-old woman presented to us in December, 2004 to undergo living related donor renal transplantation, her sister being the donor. She had suffered from hypertension for 20 years and had frequent first trimester abortions. She had renal failure in 1993 in the postpartum period and renal biopsy was not attempted at that time in view of the deranged coagulation parameters. She had severe bleeding following hysterectomy in 2002; her renal function status was not known at that time. Renal replacement therapy was initiated, first, hemodialysis and then, continuous ambulatory peritoneal dialysis (CAPD) in November 2003. She had one episode of CAPD peritonitis in January 2004.

On examination, she was found to be clinically stable and her blood pressure was under good control with antihypertensive medications. Pretransplant work-up revealed: 2+ proteinuria with occasional red blood cells in the urine sediment; VDRL nonreactivity; antinuclear antibody positive, no double-stranded DNA negative; prolonged aPTT not correctable with control plasma; thrombocytopenia with normal bleeding time; anticardiolipin IgA, IgG, IgM (titers very high for IgG); shrunken kidneys in an ultrasonogram, and viral serology negative for HBsAg, antiHCV, and antiHIV antibodies. She was diagnosed with antiphospholipid antibody syndrome of unknown etiology.

She underwent renal transplantation in 2005, azathioprine and cyclosporin A (CsA) immunosuppressive regime and low molecular weight heparin (LMWH) for the prevention of graft thrombosis in view of the presence of antiphospholipid antibodies. She had good diuresis in the posttransplant period.

The patient had developed thrombocytopenia—82,000 per cubic mm preoperatively to 30,000 per cubic mm on day 3. The lactate dehydrogenase (LDH) level was elevated (479 U/L) and there were no fragmented red blood cells in a peripheral smear. We considered CsA-associated hemolytic uremic syndrome (HUS) as one of the differential diagnoses because the patient's thrombocytopenia was associated with a drop in Hb levels (8.3 g/dL on day 2 to 6.5 g/dL) and with elevated LDH. LMWH and CsA were stopped; mycophenolate mofetil (MMF) was substituted for azathioprine. She had unexplained diffuse abdominal pain on day 3 which persisted for a few days thereafter with some improvement following the administration of narcotic analgesics. As there was a steady improvement in renal function despite worsening thrombocytopenia with no evidence of systemic HUS, CsA was restarted on Day 4. Serum creatinine levels reached a nadir of 1.8 mg/ dL on day 5.

She had a drop in urine output but a rise in serum creatinine on day 6. Cyclosporin was stopped again in view of the worsening renal function. Serial ultrasonography of the graft showed a progressive rise in the resistivity index although there was good flow in the renal vessels. She was pulsed with methylprednisolone from day 7 onwards for three days and graft renal biopsy was done the next day after platelet transfusion. Serum creatinine levels reached a peak of 2.9 mg/dL on day 9. The serial lab values of serum creatinine and platelet count are entered in Table 1.

**Table 1:** Laboratory investigation reports and consequent treatment decisions

Postop day	Serum creatinine (mg/dL)	Platelet count (per cubic mm)	Remarks
3	2.1	30,000 (preop 82,000)	CsA, Azathioprine, and heparin stopped; MMF started
4	1.9	22,000	4 units of platelets transfused; CsA restarted as GFR continued to improve
5	1.8	45,000	
6	2.1	48,000	CsA stopped as GFR started declining
7	2.3	45,000	4 units of platelets transfused, pulsed with methyl prednisolone, graft kidney biopsy done the next day
9	2.9	87,000	
11	2.7	1,25,000	

Renal histopathology using light microscopy showed features of thrombotic microangiopathy in glomerular capillaries; there was no evidence of acute cellular rejection.

**Figure 1:**
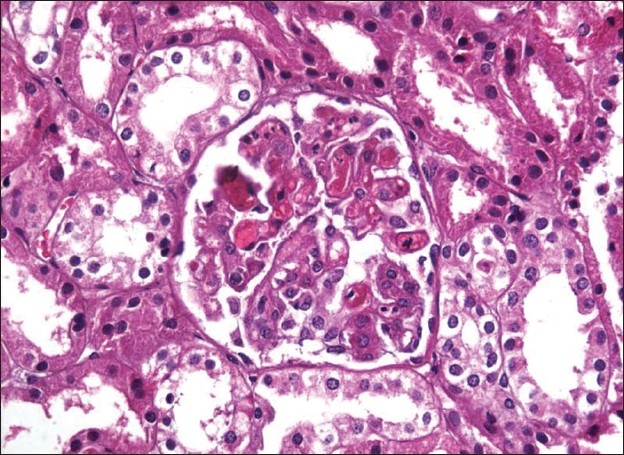
Transplant kidney biopsy under light microscopy showing capillary luminal thrombosis in the glomerulus; tubules and interstitium were normal. (H & E stain ×400)

Tubules and interstitium were normal and there was no dilatation of the peritubular capillaries. Immunofluorescence showed mesangial IgM deposits [Figure 1]. The patient was maintained on prednisolone and MMF and LMWH was restarted followed by oral anticoagulation.

At the time of discharge on day 12, the patient was clinically better although serum creatinine levels were still high (2.5 mg/dL) and there was transaminitis (SGOT: 79 U/L, SGPT: 184 U/L; Bilirubin 1.0 mg/dL (0.4 mg/dL); no hepatitis C viral RNA). There was significant clinical and biochemical improvement on follow-up thereafter (platelet count: 266000 cells/cubic mm, serum creatinine: 1.3 mg/dL, SGOT/SGPT: 94/42 U/L six weeks later). Liver enzymes became normal only in the fifth month.

The patient had posttransplant diabetes mellitus three months after transplantation and three episodes of urinary tract infection (UTI), probably related to the vesicoureteric reflux (VUR) into the graft (micturating cystourethrography as part of the urological evaluation for recurrent UTI revealed grade 5 VUR into the graft kidney). She is doing well now more than two years posttransplant with a serum creatinine level of 1.1 mg/dL in the last follow-up visit a month ago. Immunosuppression therapy at the last visit consisted of Prednisolone 10 mg once a day and MMF 750 mg twice a day. The patient is also on acitrom 3 mg and 4 mg on alternating days and bedtime cephalexin for UTI prophylaxis.

## Discussion

Thrombotic microangiopathy (TMA) is one of the causes of renal dysfunction in the posttransplant period. The incidence of the disease appears to be remarkably higher in transplant patients than in the general population, probably because of the clustering of several risk factors in this particular group of patients.[1] Posttransplant thrombotic microangiopathy has been reported with varied incidence ranging from 3 to 14% with calcineurin inhibitor (CNI)-based immuno suppression.[2] Posttransplant TMA can be *de novo* (occurring for the first time) triggered usually by: i) a CNI-based immunosuppressive regimen and less frequently by ii) viral infections (CMV, Parvovirus, Hepatitis C virus etc)[3,4] apart from iii) severe acute vascular rejection or iv) a recurrence of the primary disease, hemolytic uremic syndrome/thrombotic thrombocytopenic purpura (HUS/TTP) in the allograft and can complicate antiphospholipid antibody syndrome (APLA).

In an analysis of USRDS the incidence of TMA in renal transplant recipients was noticed to be 5.6 episodes per 1000 person-years. The risk was highest for the first three months after the transplant and the risk factors included the female gender, age (youth and the elderly), and the initial use of sirolimus. *De novo* TMA is much less common than recurrent HUS.[5] In view of the distinct characteristics and clinical courses, it has been suggested to classify posttransplant TMA into localized and systemic forms. Systemic form is TMA associated with thrombocytopenia and microangiopathic hemolysis. Patients with systemic TMA have a greater rate of graft loss.[2]

When thrombotic microangiopathy occurs in renal allografts, it is prudent to discontinue CNI and switch over to alternative immunosuppression, especially when the microangiopathy is localized.[2,6] When thrombotic microangiopathy complicates APLA syndrome, the patient has to be heparinized and maintained on lifelong oral anticoagulation.[7]

The various renal syndromes in primary antiphospholipid antibody syndrome include ARF secondary to TMA, renal vein thrombosis, cortical necrosis, renal infarction, and catastrophic antiphospholipid syndrome; renovascular hypertension; proteinuria—modest to nephrotic; glomerular diseases such as mesangial IgA deposits, membranous nephropathy; progressive chronic renal failure, and thrombosis in renal allografts.[7]

Our patient presented with end stage renal disease (ESRD) in association with thrombocytopenia, a positive lupus anticoagulant test, and high titers of anticardiolipin antibodies. There was no conclusive evidence for systemic lupus erythematosus (SLE). The cause of antiphospholipid syndrome is not clear as she was not investigated completely in the initial phase and had come to us late in the course of her renal disease. She was started on CNI-based immuno suppression along with LMWH to minimize the risk of graft thrombosis.

The patient had thrombocytopenia as the first manifestation in the first week after the transplant. We stopped all the drugs that could cause thrombocytopenia (CysA and LMWH). As there was no definite evidence for hemolysis and her serum creatinine levels were progressively declining then, the suspicion for TMA was low and CysA was restarted. With the decline in renal function three days later, our differential diagnoses included TMA and acute cellular rejection. Cyclosporin A was stopped again and anticoagulation restarted once the diagnosis became clear following renal biopsy.

The overall clinical picture strongly suggests that her basic renal disease is thrombotic microangiopathy due to primary antiphospholipid antibody syndrome, first manifesting in 1993 as postpartum renal failure, subsequently progressing to ESRD and the present episode, a recurrence of TMA. Recurrence is more likely with TMA secondary to familial and atypical forms of HUS and is associated with significant risk of graft loss. SLE is unlikely to be the cause of antiphospholipid antibody syndrome in this case in view of the absence of other features of lupus.

Cyclosporin A could have contributed to the posttransplant event as there was no worsening of renal function subsequent to its withdrawal. Cyclosporin A has been observed to cause TMA, more often in a localized form, requiring graft renal biopsy for diagnosis. Conversion to tacrolimus has been found to be beneficial in a retrospective review from the Tulane Medical University Center.[6]
